# Mushroom By-Products as a Source of Growth Stimulation and Biochemical Composition Added-Value of *Pleurotus ostreatus*, *Cyclocybe cylindracea*, and *Lentinula edodes*

**DOI:** 10.3390/foods13172789

**Published:** 2024-09-01

**Authors:** Gaia Carminati, Michele Di Foggia, Luca Garagozzo, Alessandra Di Francesco

**Affiliations:** 1 Department of Agriculture, Food, Environmental and Animal Sciences, University of Udine, 33100 Udine, UD, Italy; carminati.gaia@spes.uniud.it; 2 Department of Biomedical and Neuromotor Sciences, University of Bologna, 40127 Bologna, BO, Italy; michele.difoggia2@unibo.it (M.D.F.);

**Keywords:** *A. bisporus*, *C. cylindracea*, spent mushroom, FT-IR, β-glucans

## Abstract

Spent mushroom substrates (SMSs) and mushroom basal bodies (MBBs) are significant by-products because of their nutrient content even after harvesting. This study aimed to evaluate the effect of these two by-products, derived from *Agaricus bisporus* (Ab) and *Cyclocybe cylindracea* (Cc) cultivation, as potential growth and biochemical composition add-value enhancers of edible mushroom mycelia such as *Pleurotus ostreatus*, *C. cylindracea*, and *Lentinula edodes*. Fungal growth substrates enriched with SMS and MBB extracts significantly affected the growth of mushroom mycelia. In particular, on *P. ostreatus*, the MBBs Ab and Cc extracts determined an increase in mycelial weight by 89.5%. Also, by-products influenced mushrooms’ mycelial texture, which appeared more floccose and abundant in growth. FT-IR analysis showed that *L. edodes* mycelium, grown on MBB substrates, showed the highest increase in bands associated with proteins and chitin. Results demonstrated that mushroom by-products enhance mycelial growth and confer an enrichment of compounds that could increase mycelial resistance to pathogens and make a nutraceutical improvement.

## 1. Introduction

The worldwide increase in food production and the accumulation of organic waste is emerging as a growing concern [1]. Improper disposal of such residues not only incurs waste management costs but also represents the depletion of convenient assets [2,3]. Concerning edible mushroom production, SMS and some parts of the same mushrooms are significant by-products because of their nutrient content even after harvesting [4]. The market value of edible mushrooms was projected to rise to USD 62.19 billion for the year 2023 [5], with global production estimated to reach 20.84 million tons by 2026 [6]. Accordingly, edible mushroom production generates large quantities of by-products, assessed to an amount of 5 kg of wet by-product per kg of fresh mushrooms [7]. In Europe, the production of *A. bisporus* alone was already generating over 3.5 × 10^6^ t of SMS in 2013 [8]. Given the quality and quantity of this by-product, exploring new ways to utilize it could have both environmentally and economically substantial impacts. As reviewed by Leong et al. [9], edible mushroom by-products’ possible applications were explored in terms of use as substrates for new cultivation cycles of mushrooms, as biofertilizer and soil amendment, animal feed, renewable energy production source, and as bioremediation solution. In this work, we aimed to evaluate the effect of two by-products, derived from *A. bisporus* and *C. cylindracea* cultivation, as potential growth and nutraceutical add-value enhancers of the edible mushroom mycelia of *P. ostreatus*, *C. cylindracea*, and *L. edodes*. In effect, SMS, which are lignocellulosic by-products of mushroom cultivation, are not degraded completely, so they usually contain high organic matter with low macro- and micro-nutrient concentrations [10]. These substrates could be useful for non-industrial mushroom production, such as *P. ostreatus* and *L. edodes* [11]. Although some studies are already available on the use of SMS of *A. bisporus* in new mushroom growth circles [12,13,14], to the best of our knowledge, there is no news in the literature about the use of SMS from *C. cylindracea* for the same purpose nor about the use of the by-product represented by MBBs discarded before sending the product to distribution.

It is known that mushrooms are rich in bioactive molecules, mainly belonging to polysaccharides, proteins, terpenes, phenolic compounds, and unsaturated fatty acids, which can be reused in agricultural production processes.

## 2. Materials and Methods

### 2.1. SMSs and MBBs

SMSs and MBBs were provided by “Consorzio Funghi Treviso” (Istrana (TV), Italy), during spring 2023. Samples comprised cultural residues of button mushroom (*A. bisporus*, Ab) and poplar mushroom (*C. cylindracea*, Cc) free of fungal pathogens contamination. The first was produced on string beds of straw covered in peat, while the latter was cultivated on a mixture of straw and seeds pressed inside plastic boxes (20 × 35 × 55 cm^3^). The by-product sampling was carried out in two different farms, both in the province of Treviso, each specializing in the cultivation of *A. bisporus* and *C. cylindracea*, respectively. The by-products were sampled from three different batches of production.

### 2.2. Active Filtrates Extraction

**Figure 1:**
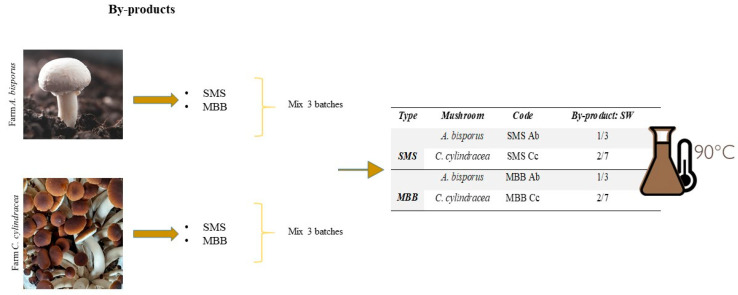
By-products and relative sterile water (SW) ratio for obtaining the extracts. SMSs and MBBs stand for spent mushroom substrate and mushroom basal body, respectively; Ab for *A. bisporus* and Cc for *C. cylindracea*.

To obtain active filtrates, samples from three different batches and belonging to each respective by-product were combined. SMSs and MBBs were blended with a variable amount of sterile water (SW), depending on the substrate (Figure 1). To obtain the extracts, by-products were inserted in sterile flasks with the relative amount of sterile water (SW) on a heated stirrer at 90 °C for 2 h.

After the extraction, the aqueous phase was filtered through two layers of sterile cloth, centrifuged at 4000 rpm for 15 min, and filtered again, as reported above.

### 2.3. In Vitro Experiments

*P. ostreatus* (Italspawn, Italy), *C. cylindracea* (Italspawn, Onigo (TV), Italy), and *L. edodes* (Soc France, France), consisting of sterile millet seeds colonized by the mycelium, were grown on PDA for 15 days at 25 °C before the experiment. Each of the extracts from SMSs and MBBs (1000 mL) was autoclaved together with 39 g/L of PDA (Potato Dextrose Agar) (Oxoid, Basingstoke, UK) and subsequently distributed in sterile Petri dishes (20 mL). Control was realized by the same concentration of PDA in 1000 mL of distilled SW instead of the extracts. Each species’ mushroom mycelial plugs (6 mm diameter) were inoculated in the center of the plates prepared as described above. The plates were incubated at 25 °C. Using a ruler, the mushrooms’ mycelial growth was noted after 5, 8, 11, 13, and 15 days post inoculation (dpi).

After 15 dpi, the main characteristics of mushroom mycelial morphology as texture (cottony or floccose), density (high, regular, or low), color (off-white, white, or pale pink), and growth (scarce, regular, or abundant) were detected by visual observations as reported by Sobal et al. [15] and described in detail in Supplementary Table S1.

The mycelium of each mushroom species was scraped at 15 dpi using a sterile blade, being careful not to pick up agar. Each plate’s mycelium was weighted using a precision balance (Engineering, Italy). The sample unit consisted of 3 plates for each mushroom and substrate. The control was represented by mycelia grown on PDA. The experiment was performed three times.

### 2.4. FT-IR Analyses of Mushrooms’ Mycelial Grown on SMSs and MBB Substrates

Mushroom mycelium, grown on SMSs and MBB substrates, was collected in sterile tubes (2 mL), stored at −80 °C and lyophilized by freeze-drying (FD-10 Freezing Dryer, Lab kits, H.K.) under vacuum (<20 Pa) at a temperature of −36 °C and freeze dried for 3 days, and stored at −20 °C until use. Mushroom samples were analyzed by FT-IR spectroscopy to obtain a rapid biochemical characterization of their main molecular components after homogenization by vortex stirrer. Infrared spectra were recorded with a FT-IR spectrophotometer (IR-TRACER-100, Shimadzu, Tokyo, Japan) equipped with an attenuated total reflectance (ATR-Diamond crystal) apparatus. The spectra were collected from 5000 to 400 cm^−1^ and averaged over 100 scans (resolution = 4 cm^−1^); three spectra were measured for each sample. Spectra were registered on mushrooms’ mycelial growth on SMSs and MBB substrates and on PDA to evaluate the potential different biochemical compositions and their variations between the treatments.

### 2.5. Data Analysis

To compare the growth rates reached at 15 dpi from each cultivated mushroom (*P. ostreatus*, *C. cylindracea*, and *L. edodes*) on each substrate (SMS: Ab and Cc; MBB: Ab and Cc; PDA), analysis of variance (ANOVA) was performed on normalized data. Normalization was performed using a Box-Cox transformation on each set of data (one for each cultivated mushroom), and normality was then tested (Shapiro–Wilk normality test) on each data group, defined as the total of the biological replicas of one mushroom species grown on one substrate. Homoscedasticity was verified by the Levene Test for each variable and a combination of them (cultivated mushroom and substrate). The error bars were calculated based on standard deviations. After performing the variance analysis, a means comparison was determined by Tukey’s test (*p* ≤ 0.05). All the statistical analyses were performed using R studio software v4.0.2 [16].

## 3. Results

### 3.1. Mycelial Growth on SMSs and MBB Substrates

**Figure 2:**
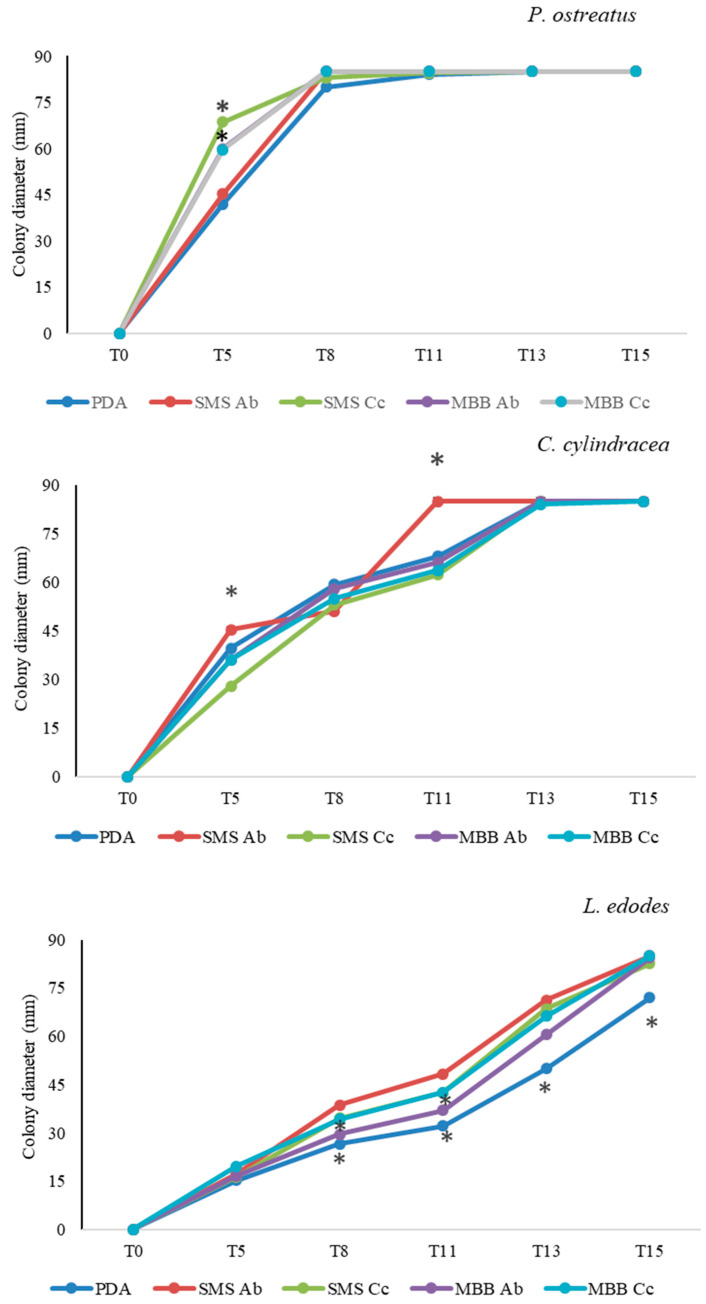
Mushroom by-products (SMS and MBB; *A. bisporus* and *C. cylindracea*, Ab and Cc) effect on the colony diameter growth (mm) of *P. ostreatus*, *C. cylindracea*, *L. edodes* mycelia. Mean values of mycelial growth were calculated among the three replicates per time. The asterisks indicate significant differences according to Tukey′s test (*p* ≤ 0.05) in comparing the growth diameters (mm) of the same mushroom on different media at the same time (T0, T5, T8, T11, T13, T15 = 0, 5, 8, 11, 13, 15 days post inoculation.

Substrates containing the different mushroom by-product extracts displayed different effects on the growth of the three selected mycelial mushroom species. Figure 2 reported the diameters of the mycelial mushroom colonies′ growth on the target by-products. A significant increase in mycelial growth was detected at 5 dpi for *P. ostreatus* on media obtained with SMSs and MBBs derived from *C. cylindracea*. The increase in the colony diameter compared to the control was by 63.4% and 41.9%, respectively. Regarding *C. cylindracea*, the colony growth was significantly stimulated at 5 and 11 dpi by the SMS Ab by-product, increasing 14.4% and 25%, respectively, compared to the control. Constant colony growth on time was registered for *L. edodes*, where all the by-products showed an active stimulation of the colony growth, especially at 13 and 15 dpi. By-product MBB Ab was slightly less active compared to the others.

**Figure 3:**
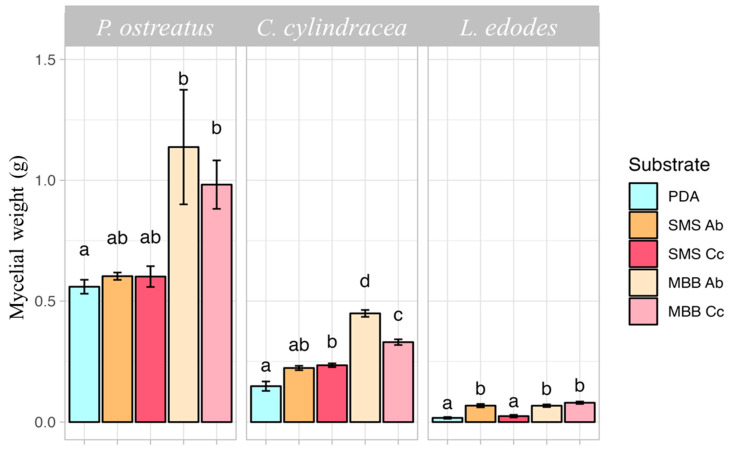
Effect of SMSs and MBBs both obtained from *A. bisporus* (Ab) and *C. cylindracea* (Cc) on the mycelial weight (g) of *P. ostreatus*, *C. cylindracea*, and *L. edodes* after 15 days of incubation at 25 °C. Mean values of mushroom weights were calculated among the three replicates per substrate. Different letters indicate significant differences according to Tukey′s test (*p* ≤ 0.05).

Regarding the mycelial weight (Figure 3), in the case of *P. ostreatus*, the MBBs Ab and Cc substrates determined a significant increase of 89.5% (on average) compared to the control. The most effective substrate for the growth of *C. cylindracea* was MBB Ab, followed by MBB Cc, with both displaying a weight increase more than double that of the control. However, with this mushroom mycelium, a significant increase in weight of 57.8% was also registered by using SMS Cc extract. The findings on *L. edodes* highlighted how most of the extracts, except for SMS Cc, positively impacted the growth of the mycelium weight with a significant increase compared to the control.

**Figure 4:**
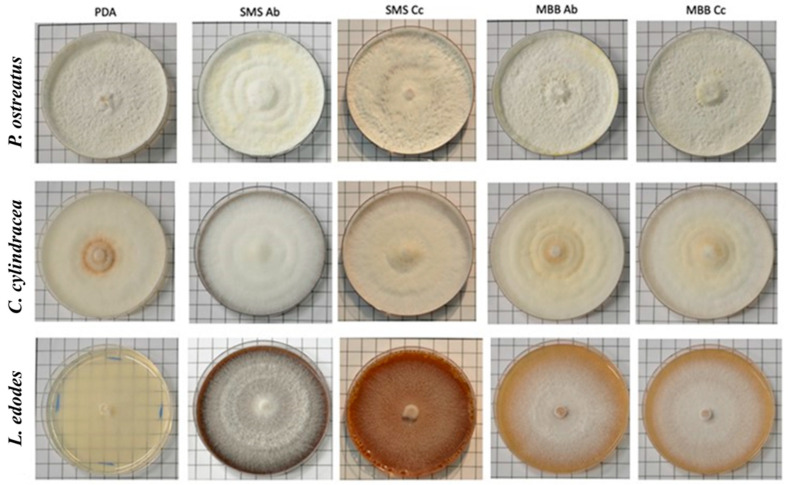
Observations of mycelial growth of *P. ostreatus*, *C. cylindracea*, and *L. edodes* grown on PDA (control) and PDA amended with extracts obtained from SMSs and MBBs of *A. bisporus* (Ab) and *C. cylindracea* (Cc). The images of the mushrooms′ mycelial growth were obtained after 15 days of incubation at 25 °C.

Table 1 reports the main mycelial characteristics of all three mushroom species grown on the five different substrates, shown in Figure 4. The by-products present in the media seemed to influence mainly the mycelial texture and growth. In particular, *C. cylindracea* growing on the SMS and MBB by-products displayed a floccose texture and abundant growth. A high density was also detected for *P. ostreatus* and *L. edodes* grown on SMS Ab.

### 3.2. FT-IR Analysis on Mushrooms′ Biochemical Composition

IR spectroscopy demonstrated enormous potential in characterizing mycelial mushrooms’ biochemical composition [17,18,19,20,21], giving insight into their biochemical composition. Figure 4 shows the IR spectra of *L. edodes* cultivated on different substrates, i.e., the species showing the most evident differences in its biochemical composition (and consequently on IR spectra). This spectroscopic evidence can be related to the finding that *L. edodes* was the only mycelial mushroom showing an increase in colony diameter (Figure 2) and weight (Figure 3) compared to the control. All IR spectra are dominated by bands attributed to polysaccharides (mainly chitin and beta-glucans) and proteins, as previously reported for other *Basidiomycetes* fungi [17]. More in detail, bands at 1635, 1558, 1543, 1406, 1256, and 1245 cm^−1^ were attributed to proteins; bands at 1635, 1543, 1451, 1377, 1320, 1147, 1073, and 1026 cm^−1^ to chitin; bands at 1719 and 1406 cm^−1^ to lipids. Most of these bands showed an increased intensity compared to *L. edodes* mycelium grown on PDA, thus indicating an increased content of these biochemical compounds. In the case of mycelium grown on SMS Cc, some proteins′ bands showed a shift in their wavenumber, such as the Amide II and III bands at 1558 and 1256 cm^−1^. Those bands are sensible to protein structural variations; in particular, both shifted towards lower wavenumbers, indicating a decrease in α-helix structure and an increase in random and β-sheet structures, which can be caused by a high protein content [17]. Glucan bands are often superimposed on chitin bands because of their polysaccharidic origin. Nevertheless, it is worth noting that IR bands at 952, 930, and 895 cm^−1^, indicative of the presence of alpha and beta-glucans [17] were particularly enhanced in *L. edodes* mycelium grown on MBB Cc. The diagnostic bands of hemicellulose derivatives (mannan at 798 cm^−1^ and galactan at 767 cm^−1^) were not affected by the different substrates [22].

## 4. Discussion

The present study′s findings highlighted a promising application for the two different wastes generated by mushroom crops. While MBB constitutes a relatively small fraction of the overall waste from mushroom productions, SMS represents an important waste successfully used as biofertilizer and soil amendment [23,24,25]. Therefore, MBB could represent a by-product of special interest that opens up an innovative application for this specific waste material. Actually, most of the studies are concentrated solely on the application of SMS directly after mushroom production. Consequently, there is a notable absence of information that comprehensively comprehends the impact of mushroom waste as a promising by-product. The obtained results showed that mushroom growth on substrates enriched with SMS and MBB extracts from two different mushrooms, *A. bisporus* and *C. cylindracea*, significantly affected the growth of the cultivated mycelium. The weight increase observed in the mycelial mushrooms cultivated on MBB-enriched substrates was particularly noteworthy in the present study.

Moreover, there is a lack of studies specifically addressing the use of MBB as a by-product. Despite being a minor waste component, it encapsulates a concentration of nutrients that would not be overlooked. In fact, applying extracts in mushroom crops could improve the nutraceutical properties of mushroom fruit bodies, which are deeply influenced by the cultivation substrate [26]. In effect, fungi, including *A. bisporus* and *C. cylindracea*, are known to produce secondary metabolites such as polysaccharides, lipopolysaccharides, essential amino acids, peptides, glycoproteins, triterpenoids, and fatty acids, often investigated for their potential as plant growth stimulation [25]; such activity could account for the increased growth of mushroom mycelia on MBB substrates.

**Figure 5:**
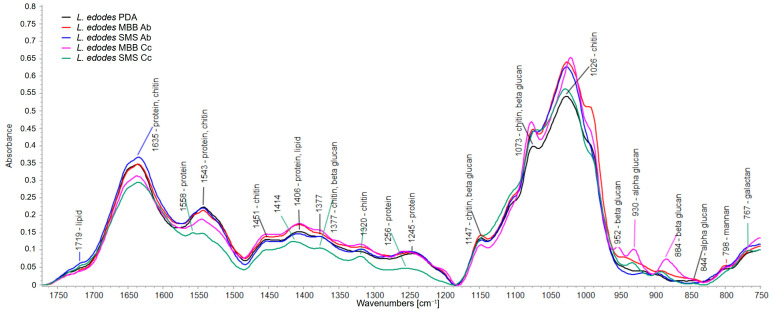
IR spectra of *L. edodes* mycelium grown on different substrates with the band’s attribution.

*L. edodes* grown on MBB substrates showed the highest increase in bands associated with proteins and chitin (such as Amide I, II, and III bands, as reported in Figure 5), which indicates enrichment of these compounds in the mycelium with interesting nutraceutical consequences. Chitin provides mechanical reinforcement to mushrooms, which may also increase their resistance to pathogens [20]. Moreover, chitin extraction from mushrooms is gaining increasing interest because it is free from potential allergens and requires no demineralization (both problems that affect chitin extraction from crustaceans) [27]. The MBB Cc substrate also showed an interesting increase in the bands diagnostics of alpha (930 cm^−1^) and beta-glucans (952 and 884 cm^−1^), of which *Basidiomycete* mushrooms are rich [17,22]. Interestingly, the least growth-promoting substrate for *L. edodes* was SMS Cc, which showed a general decrease in the IR intensity in the 1750–1200 cm^−1^ spectral region. This decrease may indicate a lower protein, chitin, and lipids content. However, the shift towards higher wavenumbers of the Amide II (from 1543 to 1558 cm^−1^) and Amide III (from 1245 to 1256 cm^−1^) bands could indicate either a conformational variation of proteins (from α-helix to random and β-sheet structure) or enrichment of protein compared to chitin [17], which leads to a not significant weight increase in *L. edodes* mycelium cultivated on SMS Cc substrate (Figure 4). These observations can lead to interesting nutraceutical considerations: a high protein content is preferable for culinary purposes, while the presence of glucans can have dietary and medical significance [17,20,22,28,29]. The results demonstrated substantial significance in mycelial growth, but whether the observed increase correlates with a proportional enhancement in basidiocarp production remains uncertain. While mycelium growth has been observed to establish favorable conditions for the production of fruiting bodies [30], a short timeframe for the spawn to complete substrate colonization does not correspond with high mushroom yield, as reported by Noonsong [31]. However, faster growth would reduce the impact of mushroom diseases during cultivation, such as green mold and bacterial infections [32].

## 5. Conclusions

The research evaluated the use of SMS and MBB by-products as potential growth and nutraceutical add-value enhancers of *P. ostreatus*, *C. cylindracea*, and *L. edodes* mycelia. This study evaluated for the first time the possibility of using these by-products with an interest related to cultivated mushroom growth promotion together with nutraceutical purposes. The extracts showed different activity depending on the mushroom species. In particular, the extracts obtained from mushroom basal bodies showed good weight gain in all three mushroom mycelia.

Also, FT-IR spectra suggested that by-products, in particular MBB, could potentially be used as *L. edodes* implementers of proteins and chitin, a functional aspect, also related to a potential prevention of mushroom pathogens attack.

However, additional research, in small-scale experimental trials, is needed to elucidate the application and the effects of the different by-products within mushroom cultivation.

## Figures and Tables

**Table 1:** Mushrooms’ mycelial characteristics on different growth substrates.

			Mycelial Characteristics			
Mushroom	Substrate	Texture	Density	Growth	Aerial Hyphae	Color
	PDA	Cottony	++	Regular	Regular	White
	SMS Ab	Cottony	+++	Regular	Abundant	Off-white
*P. ostreatus*	SMS Cc	Floccose	++	Regular	Regular	White
	MBB Ab	Cottony	++	Regular	Regular	White
	MBB Cc	Cottony	++	Regular	Regular	White
	PDA	Cottony	++	Regular	Regular	Off-white
	SMS Ab	Floccose	+++	Regular	Abundant	White
*C. cylindracea*	SMS Cc	Floccose	++	Regular	Abundant	Off-white
	MBB Ab	Floccose	+++	Regular	Abundant	Off-white
	MBB Cc	Floccose	+++	Regular	Abundant	Off-white
	PDA	Cottony	+	Irregular	Scarce	White
	SMS Ab	Floccose	+++	Irregular	Abundant	White
*L. edodes*	SMS Cc	Cottony	+	Irregular	Scarce	Off-white
	MBB Ab	Cottony	++	Irregular	Regular	White
	MBB Cc	Floccose	++	Irregular	Regular	White

+++ = High density. ++ = Regular density. + = Low density.

## Data Availability

The original contributions presented in the study are included in the article, further inquiries can be directed to the corresponding author.

## Supplementary Materials

The following supporting information can be downloaded at: https://www.mdpi.com/article/10.3390/foods13172789/s1, Table S1: *Pleurotus*, *Cyclocibe* and *Lentinula* genera: mycelial mushroom morphological characteristics described by Sobal et al., 2007 [15]. Mycelia were grown on PDA at 25 °C.
