# Supplementary material for: Mushroom By-Products as a Source of Growth Stimulation and Biochemical Composition Added-Value of Pleurotus ostreatus, Cyclocybe cylindracea, and Lentinula edodes
Source: Foods. 2024 Sep 1;13(17):2789. doi: 10.3390/foods13172789 (PMC11395502; doi:10.3390/foods13172789)
Supplement: Supplementary file 1 [file foods-13-02789-s001.zip › foods-3162557-supplementary.pdf]

Supplementary Table S1. *Pleurotus, Cyclocibe* and *Lentinul*a genera: mycelial mushroom morphological characteristics described by Sobal et al., 2007 [15]. Mycelia were grown on PDA at 25 °C.

	**Texture**	**Density**	**Growth**	**Aerial hyphae**	**Color**
Pleurotus	Cottony<br>Velvety	Regular<br>High	Regular<br>Irregular	Abundant<br>Regular	White<br>Pinkish White
Cyclocibe	Velvety<br>Floccose	Regular<br>Low	Regular<br>Irregular	Abundant<br>Regular<br>Scarce	White<br>Off-White
Lentinula	Velvety	High	Irregular	Regular	White
